# Closing the gap to achieve HBV elimination by 2030: A global pursuit fueled by regional successes

**DOI:** 10.1016/j.lanwpc.2021.100254

**Published:** 2021-08-25

**Authors:** Robert J. Wong

**Affiliations:** 1 Division of Gastroenterology and Hepatology, Stanford University School of Medicine, Stanford. CA; 2 Gastroenterology and Hepatology Section, Veterans Affairs Palo Alto Healthcare System, Palo Alto, CA

In *The Lancet Regional Health – Western Pacific,* Jidong Jia and colleagues report on the improvements in linkage to care, disease monitoring and testing, and antiviral treatment among adults with chronic hepatitis B virus (HBV) infection in Beijing, China [1]. The investigators analyzed patients using the Basic Medical Care Insurance for Employees (BMCIE) database from 2010 to 2018. The BMCIE is a mandatory social insurance program that includes both employed and retired adults and covers nearly 80% of the total population in Beijing. From 2010 to 2018, the proportion of patients with chronic HBV linked to care increased from 4.77% to 16.61%, the proportion undergoing HBV laboratory monitoring increased from 4.41% to 16.39%, and the proportion of treatment-eligible chronic HBV patients receiving antiviral therapy improved from 3.92% to 30.88%. While there is still room for improvement, these trends are encouraging for the progress towards the World Health Organization's Global Health Sector Strategy on viral hepatitis elimination by 2030, defined as a reduction in i) hepatitis-related deaths by 65% and ii) new chronic viral hepatitis infections by 90% [2,3].

Investigators from the Polaris Observatory Collaborators have previously highlighted the significant gaps in diagnosis and linkage to care and treatment among chronic HBV patients globally. Among the estimated 292 million individuals with chronic HBV, only 10% were diagnosed and linked to care, and among the estimated 94 million individuals with treatment-eligible chronic HBV, only 5% had received antiviral therapy [4]. While some regional heterogeneity exists in rates of diagnosis/linkage to care and antiviral treatment, it is particularly concerning that world regions with the greatest prevalence of chronic HBV (central, east, and southeast Asia, sub-Saharan Africa) have some of the lowest rates of HBV diagnosis and HBV treatment. Progress towards global elimination of viral hepatitis requires a coordinated action plan that engages stakeholders from policy makers and public health advocates to clinicians and researchers. Chief among these is the importance of government investment in the development of a national viral hepatitis strategy as well as dedicated funding to support the success of such initiatives. In a recent study by Smith et al, investigators surveyed the Ministries of Health of 194 Member States of the WHO in 2017 to assess progress towards viral hepatitis elimination [2]. While 63% of Member States reported developing a national viral hepatitis plan, only 37% indicated dedicated funding to support these initiatives. Despite the gaps in funding and resources to implement viral hepatitis elimination plans, it is encouraging that 69% of Member States reported having guidance on diagnostic testing for HBV, and 64% reported having guidance on referral pathways for HBV treatment and care in place [2]. Nevertheless, major gaps remain, which provide important opportunities for future research and quality improvement programs, particularly among underserved and less resourced world regions, populations where a major proportion of global chronic HBV burden remains [5].

A recent commission of international experts identified key recommendations that are needed to accelerate the global elimination of viral hepatitis [6]. The experts identified the need for innovative support and funding to ensure that national and regional viral hepatitis elimination strategy plans have adequate resources to be effectively implemented. In addition, there is a need to ensure a comprehensive approach to HBV care, from improving global access to both birth dose (and adult dose, when appropriate) HBV vaccination, innovation and access to high quality and affordable HBV diagnostics, decentralized models of HBV care that expands access to care and improves HBV linkage, particularly among underserved and vulnerable populations, and removing barriers for patients to access affordable and highly effective antiviral therapies [6]. Data from the current study by Li and colleagues report on the encouraging progress that has been achieved in Beijing, China. The global pursuit to achieve viral hepatitis elimination by 2030 will be fueled by these regional successes, and dissemination of lessons learned from region and county-specific elimination programs will be important to help global partners develop and implement their own unique strategies to progress towards viral hepatitis elimination goals.

## Declaration of competing interest

Dr. Wong receives research grants (to his institution) and serves as a consultant and advisor to Gilead Sciences.
